# Elevated stress during pregnancy in women of Turkish origin: Results from a prospective cohort study

**DOI:** 10.1093/eurpub/ckac129.608

**Published:** 2022-10-25

**Authors:** L Scholaske, S Entringer, O Razum, J Spallek

**Affiliations:** 1 Research Cluster Data - Methods - Monitoring, DeZIM-Institute, Berlin, Germany; 2 Institute for Medical Psychology, Charité - Universitätsmedizin Berlin, Berlin, Germany; 3 School of Public Health, University of Bielefeld, Bielefeld, Germany; 4 Department of Public Health, Brandenburg University of Technology, Senftenberg, Germany

## Abstract

**Background:**

Ethnic health disparities exist in the context of pregnancy and childbirth, suggesting that women of Turkish origin (i.e., they or their parents born in Turkey) in Germany have higher risks for some adverse maternal health and child developmental outcomes. Stress is believed to be a relevant pathway by which migration may be associated with these risks. In this study, we tested associations of Turkish origin with stress biology and psychological stress experiences during pregnancy.

**Methods:**

140 pregnant women (33 of Turkish/26 of other origin) participated in a prospective cohort study that was carried out in Bielefeld and Berlin (Spallek et al., 2020). Inflammatory markers CRP and IL-6 from venous blood samples and diurnal cortisol profiles from salivary cortisol samples were derived and participants completed the Perceived Stress Scale (PSS) and Center for Epidemiologic Studies Depression Scale (CESD) at two study visits during pregnancy (T1: 20-25 weeks of gestation, T2: 30-35 weeks of gestation). Multilevel models were conducted to account for the nested data structure due to repeated measurements.

**Results:**

Compared to non-migrant women, women of Turkish origin had significantly higher inflammatory levels (b = 0.28, SE = 0.14, p=.052) (Spallek et al., 2021), a blunted cortisol awakening response (b=-0.21, CI=-0.38–0.03, p<.05), a flatter diurnal cortisol slope (b = 0.02, CI = 0.00-0.04, p<.05), and higher PSS (b = 0.46, SE = 0.13, p< .001) and CESD scores (b = 0.29, SE = 0.08, p<.001) during pregnancy after adjusting for socioeconomic factors.

**Conclusions:**

The results of our study suggest higher stress at the biological and psychological level in pregnant women of Turkish origin. Stress is a risk factor for pregnancy complications and poor birth and child developmental outcomes. To reduce such unequally distributed risks, interventions for stress reduction are needed that are tailored to women of Turkish origin.

**Key messages:**

• Women of Turkish origin in Germany have elevated psychological and biological stress levels during pregnancy compared to non-migrant women.

• This finding underscores the need for targeted interventions to reduce stress in this high-risk group.

